# Social Change Communication: Need of the Hour for the Prevention of HIV/AIDS

**DOI:** 10.4021/jocmr2009.12.1285

**Published:** 2010-02-08

**Authors:** Haresh Chandwani, Rajesh Gopal

**Affiliations:** a Department of Community Medicine, Medical College, Vadodara, Gujarat, India; b Gujarat State AIDS Control Society, Ahmedabad, Gujarat, India

## Abstract

For the last three decades or so, we all have been living with the pandemic of HIV and AIDS. Human behaviour is complex; widespread behaviour changes are challenging to achieve. Understanding the dynamics of HIV transmission can not be separated from an understanding of the broader context of poverty, inequality and social exclusion which create conditions where unsafe behaviour flourishes. HIV/AIDS is not a mere health issue: its occurrence is influenced by a number of socio-economic, cultural and ecological determinants. Social change communication is an inclusive way of responding to HIV/AIDS issues. Social change communication can tackle structural drivers of the HIV epidemic, with a particular focus on the drivers of gender inequality, stigma and discrimination, and human rights violations. Social change communication is bound to emerge as the vaccine and panacea for HIV and AIDS.

**Keywords:**

HIV; AIDS; Behaviour; Social change communication

## Introduction

For the last three decades or so, since when we all have been living with the pandemic of HIV and AIDS, the delivery of effective behaviour change strategies through robust interventions has been considered pivotal to the endeavours for reversing the global HIV epidemic.

Human behaviour is complex; widespread behaviour changes are challenging to achieve; and there are important gaps in our knowledge about the effectiveness of HIV prevention. Yet the research to date clearly documents the impact of numerous behavioral interventions in reducing HIV infection. We also know that in all cases in which national HIV epidemics have reversed, broad based behaviour change were central to success [1]. It is important to recognize the fact that understanding the dynamics of HIV transmission can not be separated from an understanding of the broader context of poverty, inequality and social exclusion which create conditions where unsafe behaviour flourishes [2].

Most of the national/regional AIDS control programme managers and other stakeholders have rightly recognized the central role of behaviour change communication (BCC) in the targeted interventions for the most at risk populations, the so called core transmitter groups.

Communication is an essential element of AIDS prevention, treatment and care efforts. Historically, such efforts have been limited by a focus on getting messages out about how HIV is transmitted, with lesser attention to cultural and social contexts in which such communication occurs. These contexts often present barriers to individual behaviour change [3]. The ‘high risk groups’ in general and people practising high risk behaviour in particular are presumed to benefit most from directed efforts for ensuring behaviour change towards safer sex from the existing pattern of sexual behaviour which predisposes them to acquisition of STI/HIV/AIDS.

However, even in countries and regions with concentrated epidemic, a comprehensive response looking into all the aspects of prevention, care, support and treatment must be preferred to pursuits in piece-meals focusing on a few individuals with limited efforts for systems strengthening. Other perspectives and approaches must be considered to ensure a comprehensive and sustained response.

The AIDS activists and development professionals working for containment of HIV/AIDS perceive very clearly that HIV/AIDS (like most of the ailments) is not a mere health issue: its occurrence is influenced by a number of socio-economic, cultural and ecological determinants. Health interventions alone, therefore, cannot lead to its prevention. Its prevention requires a concerted collaborative effort from all organizations in public life through their work and programmes.

Social change communication is an inclusive way of responding to HIV/AIDS issues. Social change communication is an umbrella term involving strategic use of advocacy, media, interpersonal and dialogue based communication, and social mobilization to systematically accelerate change in the underlying drivers of HIV risk, vulnerability and impact [3]. This integrated, inclusive and multi-sectoral approach transfers the ownership of HIV/AIDS issues, including its direct and indirect causes, impact and response, to various stakeholders, including the government, the corporate sector and civil society organizations. The focus of all organizations in mainstreaming must be to adapt their core business to respond to the challenges of HIV/AIDS. Mainstreaming and inter-sectoral approach will have to be adopted by all to ensure health sector and other much needed reforms so as to make a dent in the humongous developmental challenge.

We must find innovative ways to spark community level dialogue privately and publicly among individuals and institutions so that all voices have access to the process of making decisions concerning treatment and prevention. Those most affected by HIV and AIDS must fully own or control the essential communication processes and effect policies that impact their lives [4].

## Challenges

It is strongly felt that development of perspectives and pursuits which are based on community based, community led and community owned approaches can take care of the humongous hurdles that the developing countries are facing, especially in the field of health. The needs based and evidence based planning by the community for interventions which are monitored by the community itself is a definite key to success of such endeavours and is bound to go a long way in improving the scenario of health in these countries. Health Systems Strengthening must be ensured at all costs (requiring a massive exercise of careful prioritization in the poor countries euphemistically called developing nations) but we must maintain the sustained focus on Community Systems Strengthening (CSS) fully backed by an effective social change communication. We all have to see these words beyond not just as statements (very often truisms) but as a definite writing on the wall, which has to be facilitated by all of us through very active and proactive involvements of the civil society at large to accomplish the desired outcome. Let us all ensure a committed and concerted collective action for the same.

More often than not, ‘health seeking behaviour’ has been confused with ‘healthcare seeking behaviour’. The perspective and felt needs of the community are of paramount importance. It would be a major mistake to still consider the community as 'beneficiaries' of healthcare instead of the proactive facilitators that they are and must always be seen as.

It has been established beyond doubt that socio-economic determinants play a huge role in the scenario of physical, mental, emotional and spiritual health. The community has to be empowered enough to plan for its health, to chalk out appropriate activities and to implement and monitor them with tools, almost like a social audit. As having been the experience throughout the world, in developing countries in particular, a tokenism participation of people in healthcare programming and healthcare service delivery will not take us anywhere.

Keeping the Millennium Development Goals (MDGs) in proper perspective, all stakeholders and all implementers of health programmes and service providers in particular, must appreciate that ‘Health for All by 2000’ never just meant healthcare services for all, though issues of inequity and inaccessibility still mar even that. It has to be based on dialogue, which is necessary to promote stakeholders’ participation. Such participation is needed in order to understand stakeholder perceptions, perspectives, values, attitudes and practices so they can be incorporated into the design and implementation of development initiatives [5].

People have to take charge and find out ‘*glocal*’ solutions through the localized communities acting on evidence (facilitated by evidence available throughout the Globe) in the most cost-effective manner. We have to be aware of the fact that only when communities decide to act on their own and take charge, can a difference be made. Greater Involvement of People living with HIV/AIDS (GIPA) at all levels have been the evidence that we have generated in its support.

Concerted collective action is the need of the hour (and all hours to come) to plan and work in accordance with a needs based approach on sound and indisputable evidence generated by the community itself, and not according to the mandate of some agency/funding organization or some theoretical/transplanted assumptions for replication of the ‘best practices’ with ‘proven success’ elsewhere. It can be done by giving a voice to the voiceless, by facilitating community conversations that lead to community action, and by building channels of communication between community and government [6]. Let us get over the biggest impediment, our mindsets that health is just a matter of hospitals, doctors and healthcare, in the desired way of operating accordingly. The vital need of ensuring that the people play a central role in their health in a very empowered and proactive way must be recognized, appreciated and facilitated. Non-consideration of the perspective of the community will compel us to pay a very heavy price in the time to come.

The stigma, discrimination and denial of services to the PLHIV have been the second epidemic that we all have to contain as a part of containment of the HIV pandemic. Other marginalized communities also need to be co-opted as proactive facilitators to make the endeavours relevant and effective. Communication strategy needs also to address the very real social obstacles that prevent positive change, including the position of women in society, stigma, prejudice and marginalization [7].

The biggest hurdle in the delivery of STI services of quality to the MSM/TG community is the mindset and attitude of most of the service providers. This observation is based on various programmatic and personal experiences as a programme manager for STDs and HIV for about a decade. Realization of this reality compelled us to partner with preferred private providers and to build their capacity through structured modular trainings to facilitate management of the STIs in the targeted interventions for the MSM/TG and other most at risk populations. These activities are not just another option but an absolute must to ensure effective STI service delivery outside the formal public health infrastructure and governmental hospital/health centres accessed by very few.

In an assessment of ICTC (counselling and testing services) of a very large centre affiliated to a teaching hospital in a state in South India, we came across the situation where the counselling, testing and STI care services were never accessed by the TG population, who were staying in large numbers at a stone's throw distance. The FGD with them established beyond doubts that non-inclusion of even a single TG in the clients for provided services in that large centre was directly related to the ridicule and the stigmatizing and discriminatory demeanour of the personnel/service providers; some of the clients were subjected to in the past.

Qualified doctors have been observed to just examine the genitalia of a male/female patient with no efforts to consider the oral/anal involvement, perhaps denying the existence of alternate sexual practices and thereby wishing them away. Such judgmental approach evincing an ostrich mentality directly affects the quality and reach of the much needed services for prevention, care, support and treatment of STI/HIV. Stigma, discrimination and the denial of appropriate services will add to the burden with major untoward consequences. We have to promote and protect the rights of the clients and ensure stigma free milieu for delivery of services.

At the same time promotion and protection of human rights, inter alia, necessitate full and creative utilization of all the existing legal and societal provisions. It is ironic that the law of the land which has to putatively serve the cause of benefiting all and promoting the larger interest of the society is, more often than not, in conflict with the endeavors for the prevention of acquisition of sexually transmitted infections and the containment of HIV/AIDS. Human behaviour is indeed an interesting interplay of the extant cultural, moral, ethical, legal and societal norms asserted and overzealously guarded by the powers. For example, the differences in health behaviours are often the function of culture. Therefore, culture should be viewed for its strength and not always as a barrier. The metaphorical coupling of ‘culture’ and ‘barrier’ needs to be exposed, deconstructed, and reconstructed so that new, positive cultural linkages can be forged [8].

## Way Ahead

The institutions of family, society and the administrative and legal infrastructure go to any extents to regulate the individual’s behaviour by dictating terms to compel him/her to conform to a rigid pattern of behaviour on the pretext of checking deviant behaviour in the interest of the person and the society at large. We have been bewildered on several occasions to observe the two arms of the governmental agencies working at cross purposes but both purportedly working as per the law of the land. One agency works for promotion of health and for prevention of sexually transmitted infections and HIV/AIDS in such marginalized groups, whereas the other arm of the same government may disrupt the rapport with these communities by conducting thoughtless raids and uncalled for arrests compelling the entire activity to go underground and start operating in a clandestine manner with severing of all linkages and support for health care delivery and preventive efforts.

The response to the pandemic of HIV/AIDS necessitates working specifically with the core transmitter groups in those geographical areas where the epidemic is still concentrated and is predominantly limited to the so called high risk behaviour groups. It is ironic that these groups are ‘criminalized populations’ in most of the countries in this part of the globe. There are organized /institutionalized socio-cultural barriers and deterrents which impede these populations from reaching and using the health delivery or justice delivery services. These populations are hindered in terms of access to information, prevention, care, support and treatment because of the ambivalent approach of the governmental functionaries and the society at large.

The operations are very often at loggerheads with the other department/agency. There have been myriad misadventures of throwing the baby along with the bathwater. Such provisions and actions thereon compel the CSWs, MSM and IDUs to be driven away from the HIV services besides the specific instances of discrimination and even violence at the hands of law enforcing agency with practically no safeguarding of rights from the judicial infrastructure. The concerned laws as follows, Immoral Traffic (Prevention) Act, Section 377 of Indian Penal Code and the Narcotic Drugs and Psychotropic Substances (NDPS) Act, tend to criminalize the CSW, MSM and IDU populations respectively thereby obstructing any prevention, care support and treatment interventions with them. The assertion of basic rights of behaving in accordance with one’s personal behavioral orientation and choice of livelihood options are also severely hindered by the archaic pieces of legislation, which are at best the vestiges of the colonial past. Most of such obsolete laws have already been repealed or supplanted by more progressive and rights based provisions in most of the other countries. All the stakeholders must work in complete synergy to ensure decriminalization of the concerned community/practice/profession of sex work. The need of the hour is writ large as the writing on the wall, namely, it is high time we repeal the archaic laws and facilitate concerted collective action for the containment of HIV/AIDS through a mainstreamed and multi-sectoral response which is a must for this gigantic developmental challenge. Appropriate advocacy, communication and social mobilization at all the levels in tandem through the myriad stakeholders are strongly recommended for the same.

The cited instances further corroborate the observations at the grassroots and strengthen the evidence necessitating building of a case for provision and equitable access of non-judgmental and non-stigmatizing quality services at all levels. Community has to be at the centre. Paradigm shifts are needed in approaches to HIV and AIDS. The main strategy has to be effective communication with a shift from ‘message’ to ‘voice’ and such communication can tackle structural drivers of the HIV epidemic, with a particular focus on the drivers of gender inequality, stigma and discrimination, and human rights violations [9]. It is high time we understand that some of the past failures have been wrongly blamed on the individual in total disregard to the context which shapes the individual [10].

## Conclusions

Full ownership of and active participation by a mobilized community equipped with necessary communication (and other) skills, are a must. Fully functional social networks and robust internal communication structures would be needed to facilitate interventions within the context of the empowered communities.

The effectiveness of HIV prevention has been established cutting across the nation and geographical regions only in meaningful presence of: 1) multi-dimensional prevention strategy; 2) directed efforts to supplement the limited impact of individual BCC; 3) effective addressing of the social, economic, cultural, environmental and other determinants of health development.

Social change communication is bound to emerge as the vaccine and panacea for HIV and AIDS.
